# Changes in the Nasal Colonization with Methicillin-Resistant *Staphylococcus aureus* in Children: 2004-2009

**DOI:** 10.1371/journal.pone.0015791

**Published:** 2010-12-29

**Authors:** Wen-Tsung Lo, Chih-Chien Wang, Wei-Jen Lin, Sheng-Ru Wang, Ching-Shen Teng, Ching-Feng Huang, Shyi-Jou Chen

**Affiliations:** 1 Department of Pediatrics, Tri-Service General Hospital, National Defense Medical Center, Taipei, Taiwan; 2 Department of Pediatrics, Wan Fang Hospital, Taipei, Taiwan; Columbia University, United States of America

## Abstract

**Background:**

*Staphylococcus aureus* is an important cause of infection, particularly in persons colonized with this organism. This study compared the annual prevalence and microbiological characteristics of methicillin-resistant *S. aureus* (MRSA) nasal colonization in Taiwanese children from 2004 through 2009. Risk factors for MRSA were determined for the overall study period.

**Methods:**

Children from birth to ≤14 years of age presenting for health maintenance visits or attending 1 of 57 kindergartens were recruited. Nasal swabs were obtained, and a questionnaire was administered. The prevalence and microbiological characteristics of MRSA colonization were also calculated for two 3-year periods: 2004–2006 and 2007–2009.

**Results:**

Cultures of the anterior nares were positive for *S. aureus* in 824 (25.8%) of the 3,200 children, and MRSA colonization was found in 371 (11.6%) children. The prevalence of *S. aureus* colonization decreased from 28.1% in 2004–2006 to 23.3% in 2007–2009 (*p*<0.01), whereas the prevalence of MRSA colonization increased from 8.1% to 15.1% during this period (*p*<0.0001). Multivariate analysis revealed that the independent risk factors for MRSA carriage were different for male and female children, and also among age groups. Most MRSA isolates belonged to sequence type 59 (ST59) (86.3%); however, a multiresistant MRSA clone with ST338 background emerged in 2007–2009. Ten (62.5%) of the 16 MRSA isolates expressed the genotypic profile ST338/staphylococcal cassette chromosome *mec* V_T_/Panton-Valentine leukocidin-positive/staphylococcal enterotoxin B-positive, and differed only in their antimicrobial susceptibility patterns.

**Conclusion:**

The prevalence of nasal colonization by MRSA increased among healthy Taiwanese children from 2004–2006 to 2007–2009, despite an overall decrease in the prevalence of nasal colonization by *S. aureus.* A multiresistant MRSA clone characterized as ST338 was identified from these children.

## Introduction

*Staphylococcus aureus* is a common human pathogen of skin infections and invasive diseases in all age groups, such as pneumonia, osteomyelitis, and endocarditis, in healthcare and community settings [1]. Methicillin-resistant *S. aureus* (MRSA) isolates have been recognized as a source of healthcare-associated infections since the 1960s [2]. Over the past decade, the traditional notion of MRSA as a pathogen that is seemingly confined to the nosocomial arena has been challenged with the emergence of community-associated MRSA (CA-MRSA) in healthy individuals without conventional risk factors for MRSA acquisition, especially in the USA [3], [4]. Reports of rapidly progressive fatal disease and serious complications resulting from virulent CA-MRSA infection including sepsis, necrotizing pneumonia, and necrotizing fasciitis have alerted medical professionals and the community alike to the need to face the increasing threat from community-based MRSA infections [5]–[8]. Initially, CA-MRSA strains were thought to lack an association with healthcare settings and to have unique microbiologic characteristics such as limited antibiotic resistance (except to β-lactam antimicrobial agents), different exotoxin gene profiles (e.g., Panton-Valentine leukocidin, PVL), and smaller staphylococcal cassette chromosome *mec* (SCC*mec*) type IV [9]. However, these epidemic strains have been shown to be transmitted in healthcare settings and to exhibit emerging resistance to non-β-lactam agents [10]–[12]. In Taiwan, MRSA strains of sequence type 59 (ST59), determined by multilocus sequence typing (MLST) and carrying type IV or a variant, type V_T_ SCC*mec* elements, were found to be the major strains of CA-MRSA [13]–[18].

*S. aureus* colonizes the anterior nares and other body sites, but the anterior nares are the most consistent site of colonization [19]. Carriage of *S. aureus*, including MRSA, is common in children, and genetic evidence supports a relationship between nasal carriage of *S. aureus* and MRSA and subsequent invasive staphylococcal infection [15], [20]–[23]. Children could act as vectors for spreading *S. aureus* and MRSA to both community and hospital environments [24]. In addition, day-care centers constitute reservoirs of MRSA where children are at increased risk of nasal colonization [15], [25], [26].

In Taiwan, the first island-wide prevalence survey of nasal colonization with *S. aureus* was conducted in 2005 and 2006, and it showed a measurable prevalence of colonization with MRSA in the community [27]. The objectives of the current study in children were to assess trends in the overall prevalence of nasal colonization by *S. aureus*, to identify the potential risk factors for MRSA nasal colonization, and to describe the evolving epidemiology of nasal colonization by *S. aureus* and by MRSA specifically.

## Materials and Methods

### Study design, population and location

This prospective observational study was conducted from 2004 to 2009 at Tri-Service General Hospital, a 1400-bed tertiary medical center in northern Taiwan. The study proposal was reviewed and approved by the National Defense Medical Center Institutional Review Board. Eligible children were 14 years of age or younger with no acute medical problem, who either presented for a health maintenance visit or attended one of 57 kindergartens in Taipei. Written informed consent was obtained from each child's parents or legal representative before nasal specimen collection or interviews. During the 6-year study period, all children who presented for health maintenance visits to our hospital were invited to participate in this study. In addition, the selection of the kindergartens from all kindergartens in Taipei was based on support for the surveillance study by the kindergartens' principals. The number of children sampled per kindergarten was proportional to the number of children attending each kindergarten. Based on a previous study [28], we estimated the rate of *S. aureus* colonization to be 30%. Therefore, we calculated that a sample size of 1100 children would be necessary to estimate risk factors for MRSA colonization, based on estimated prevalence ranging from 1% to 3%, with a 95% confidence interval ([CI] design effect, 1.5). Study participants were recruited sequentially until the estimated recruitment number was met.

### Data collection

Individual variables, demographic characteristics of the family, and medical history over the preceding 12 months, including previous hospitalization, medication history prior to receiving the screening test, and any underlying diseases correlated with *S. aureus* colonization status were obtained by interviews with the guardians immediately after consent was obtained and before swabs were collected [29].

### Nares cultures, bacterial strains and antimicrobial susceptibility testing

Nasal samples were obtained with a sterile cotton swab, placed in transport medium (Venturi Transystem, Copan Diagnostics, Corona, CA, USA), and then transported to and processed in the microbiology laboratory within 4 hours. Cotton swabs were plated on mannitol salt agar (MSA; BBL Microbiology Systems, Becton Dickinson, Company, Sparks, MD, U.S.A.). Each distinctive morphotype of mannitol-fermenting colony was selected from an MSA plate, subcultured to a trypticase soy agar plus 5% sheep blood agar plate (BAP; BBL Microbiology Systems, Becton Dickinson, Company, Sparks, MD, U.S.A.), and incubated at 37°C in a humidified incubator with 5% CO_2_. Cultures on BAPs were screened using Slidex Staph Plus (bioMérieux, Marcy l'Etoile, France). MRSA identification and antimicrobial susceptibility testing were performed according to Clinical Laboratory Standards Institute (CLSI; formerly known as the NCCLS) guidelines [30], [31]. Multidrug-resistant *S. aureus* isolates were defined as isolates resistant to three or more antimicrobial classes. All *S. aureus* isolates were frozen at –70°C for additional testing of organism characteristics.

### Polymerase chain reaction

Chromosomal DNA from three to five isolated colonies was prepared using the Puregene DNA purification kit (Gentra, Minneapolis, Minnesota, USA) as recommended by the manufacturer, with lysostaphin at 2 mg/ml and RNase at 4 mg/ml for the lysis step. The presence of the *lukS-PV* and *lukF-PV* genes encoding PVL components was determined by a polymerase chain reaction (PCR)-based method with the primer pair and thermocycler conditions reported by Lina et al [32]. The presence of known macrolide-lincosamide-streptogramin resistance genes (*ermA*, *ermB, ermC* and *msrA*) was determined by PCR according to a previously described method [33], [34]. Sequences specific for *sea* to *see*, *seg* to *sei*, *sek*, *seq*, *eta*, *etb*, and *tst*, encoding staphylococcal enterotoxins (SEA to SEE, SEG to SEI, SEK, and SEQ), exfoliative toxins (ETA and ETB), and toxic shock syndrome toxin-1, respectively, were detected using the methods described by Jarraud et al. and Diep et al [35], [36]. SCC*mec* typing was performed using a multiplex PCR strategy with sets of region-specific primers as previously described [37]. Screening for SCC*mec* V_T_ was performed with the primer and thermocycler conditions reported by Boyle-Vavra et al. and Huang et al [14], [27].

### Genotyping

Pulsed-field gel electrophoresis (PFGE) was performed using the CHEF Mapper XA system (Bio-Rad Laboratories, Hercules, CA, USA) according to a published protocol [38]. Findings were interpreted on the basis of standard criteria [39]. To identify PFGE polymorphisms, band patterns were analyzed using Molecular Analyst Fingerprinting, Fingerprinting Plus and Fingerprinting DST software (Bio-Rad Laboratories, Richmond, CA, USA). The grouping method was used to deduce a dendrogram from the matrix by the unweighted pair group method using the arithmetic averages clustering technique after calculation of similarities using the Dice correlation coefficient between each pair of organisms; the PFGE patterns were distinguished at the 80% similarity level. Some isolates of representative PFGE patterns were subjected to MLST. MLST was performed by PCR amplification and sequencing of seven housekeeping genes using primers designed by Enright et al [40]. Each sequence was submitted to the MLST database website for assignment of the allelic profile and sequence type.

### Statistical analysis

Data were entered into Microsoft Access XP software and exported into SPSS statistical software, version 10.0 (SPSS), which was used for data analyses. The categorical variables were compared using the chi-square test, Mantel-Haenszel test or Fisher's exact test. The trend in annual prevalence rates of colonization was examined using Poisson regression analysis. The prevalence and microbiological characteristics of colonization were also determined for two time periods (2004–2006 and 2007–2009) separately, to compare categorical variables between the two survey cycles. In addition, data from 2004 through 2009 were combined to provide more reliable estimates of the microbiological characteristics of and risk factors for MRSA colonization and to increase the study's power to detect statistically significant differences. To analyze the risk factors for carrying MRSA among male and female children in various age groups (<1 year, 1–5 years and >5 years of age), we used polytomous logistic regression to compare children with MRSA to those without *S. aureus* and children with MRSA to those with methicillin-susceptible *S. aureus* (MSSA). All parameters were initially determined by means of univariate analysis; those with *p* values of <0.05 and those being biologically meaningful were entered into the multivariate analysis. However, parameters with colinearity, tested by correlation matrices, were not simultaneously considered in the final model. In the multivariate analysis, stepwise model comparison was used to determine the best model. Risk factors were assessed using SAS 9.1.3 (SAS Institute, Inc., Cary, NC). All tests were two-tailed, and a *p* value of <0.05 was defined as statistically significant.

## Results

### Population characteristics

A total of 3200 children with ages ranging from 1 month to 14 years participated in this study: 1019 were recruited during health maintenance visits and 2181 were recruited from the 57 selected kindergartens. The median age of the participating children was 4.3 years, and 1602 (50.0%) were male.

### Isolation of *S. aureus* from children

**Figure 1:**
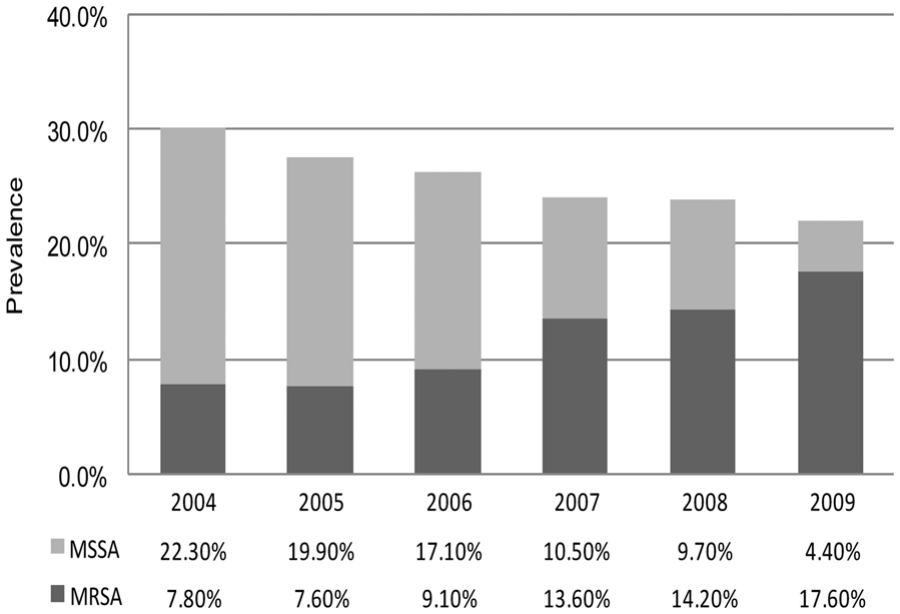
Annual prevalence of methicillin-resistant *Staphylococcus aureus* and methicillin-susceptible *S. aureus* nasal colonization in Taiwanese children, 2004–2009.

Of the 3200 nares specimens, 371 (11.6%) were positive for MRSA, 453 (14.2%) were positive for MSSA, and 2376 (74.2%) were culture negative for *S. aureus*. The percentage of MRSA isolates among all *S. aureus* isolates was 45.0%. From 2004–2006, a total of 1615 children were interviewed and examined; of these, 454 (28.1%) were culture-positive for *S. aureus*. Similarly, from 2007–2009, a total of 1585 children were interviewed and examined, and 370 (23.3%) of them were culture positive for *S. aureus*. The annual prevalence rates of MRSA and *S. aureus* nasal colonization are shown in the Figure 1. The prevalence of MRSA colonization significantly increased, at a rate of 25% per year (*p*<0.0001, test for trend), whereas the prevalence of *S. aureus* colonization decreased (*p* = 0.0006, test for trend). Moreover, the prevalence of *S. aureus* nasal colonization decreased to 23.3% during 2007–2009 (95% CI, 21.2%–25.4%; *p*<0.01) from 28.1% during 2004–2006 (95% CI, 25.9%–30.3%) (Table 1). However, the prevalence of MRSA colonization increased to 15.1% (95% CI, 13.3%–16.8%) in 2007–2009 from 8.1% (95% CI, 6.8%–9.4%) in 2004–2006 (*p*<0.0001).

**Table 1:** Prevalence of *Staphylococcus aureus* and methicillin-resistant *S. aureus* (MRSA) nasal colonization in children with different demographic characteristics, across surveys (2004–2006 vs. 2007–2009).

			Prevalence of nasal colonization, % (95% CI)			
	No. (%) of children		*S. aureus*		MRSA	
Category	2004–2006	2007–2009	2004–2006	2007–2009	2004–2006	2007–2009
All participants	1615 (50.5)	1585 (49.5)	28.1 (25.9–30.3)	23.3 (21.2–25.4)*	8.1 (6.8–9.4)	15.1 (13.3–16.8)**
Source						
Health maintenance visits	740 (45.8)	279 (17.6)	27.2 (24.0–30.4)	24.0 (19.0–29.0)	6.4 (4.6–8.1)	15.8 (11.5–20.1)**
Kindergartens	875 (54.2)	1306 (82.4)	28.9 (25.9–31.9)	23.2 (20.9–25.5)*	9.6 (7.7–11.6)	15.0 (13.1–16.9)***
Sex						
Male	822 (50.9)	780 (49.2)	28.2 (25.2–31.3)	25.6 (22.6–28.7)	8.3 (6.4–10.2)	16.7 (14.1–19.3)**
Female	793 (49.1)	805 (50.8)	28.0 (24.9–31.1)	21.1 (18.3–23.9)*	7.9 (6.1–9.8)	13.7 (11.3–16.0)***
Age, years						
<1	364 (22.5)	213 (13.5)	32.1 (27.4–36.9)	16.0 (11.0–20.9)**	6.9 (4.3–9.5)	9.4 (5.5–13.3)
1–5	720 (44.6)	503 (31.7)	23.2 (20.1–26.3)	24.7 (20.9–28.4)	7.6 (5.7–9.6)	13.5 (10.5–16.5)***
>5	531 (32.9)	869 (54.8)	32.0 (28.1–36.0)	24.4 (21.5–27.3)*	9.6 (7.1–12.1)	17.5 (15.0–20.0)**

CI, confidence interval.

*p<0.01,

**p<0.0001,

***p<0.001.

Tables 1 and 2 summarize the results of the surveillance for *S. aureus* and MRSA among children tested from 2004–2006 and 2007–2009. When prevalence was compared across surveys, a significant decrease in *S. aureus* nasal colonization occurred in children from kindergartens (*p*<0.01), in female children (*p*<0.01), and among children aged <1 year and >5 years (*p*<0.0001 and *p*<0.01, respectively). In addition, the prevalence of MRSA colonization increased in 2007–2009 among both male and female children (*p*<0.0001 and *p*<0.001, respectively), children attending both health maintenance visits and kindergartens (*p*<0.0001 and *p*<0.001, respectively), and children aged 1–5 years and >5 years (*p*<0.001 and *p*<0.0001, respectively).

**Table 2:** Prevalence of *Staphylococcus aureus* and methicillin-resistant *S. aureus* (MRSA) nasal colonization in children with different demographic characteristics, 2004–2009.

			Prevalence of nasal colonization, % (95% CI)			
	No. (%) of children		*S. aureus*		MRSA	
Category	2004–2006	2007–2009	2004–2006	2007–2009	2004–2006	2007–2009
All participants	1615 (50.5)	1585 (49.5)	28.1 (25.9–30.3)	23.3 (21.2–25.4)	8.1 (6.8–9.4)	15.1 (13.3–16.8)
Source						
Health maintenance visits^a^	740 (45.8)	279 (17.6)	27.2 (24.0–30.4)	24.0 (19.0–29.0)	6.4 (4.6–8.1)	15.8 (11.5–20.1)
Kindergartens	875 (54.2)	1306 (82.4)	28.9 (25.9–31.9)	23.2 (20.9–25.5)	9.6 (7.7–11.6)*	15.0 (13.1–16.9)
Sex						
Male^a^	822 (50.9)	780 (49.2)	28.2 (25.2–31.3)	25.6 (22.6–28.7)	8.3 (6.4–10.2)	16.7 (14.1–19.3)
Female	793 (49.1)	805 (50.8)	28.0 (24.9–31.1)	21.1 (18.3–23.9)*	7.9 (6.1–9.8)	13.7 (11.3–16.0)
Age, years						
<1^a^	364 (22.5)	213 (13.5)	32.1 (27.4–36.9)	16.0 (11.0–20.9)	6.9 (4.3–9.5)	9.4 (5.5–13.3)
1–5	720 (44.6)	503 (31.7)	23.2 (20.1–26.3)**	24.7 (20.9–28.4)*	7.6 (5.7–9.6)	13.5 (10.5–16.5)
>5	531 (32.9)	869 (54.8)	32.0 (28.1–36.0)	24.4 (21.5–27.3)*	9.6 (7.1–12.1)	17.5 (15.0–20.0)***

CI, confidence interval.

a Reference group.

*p<0.05, for comparison with reference group within survey (2004–2006 or 2007–2009),

**p<0.001, for comparison with reference group within survey (2004–2006),

***p<0.01, for comparison with reference group within survey (2007–2009).

### Questionnaire data and statistical analysis

We used polytomous logistic regression to identify risk factors for MRSA colonization by comparing children with MRSA to those with MSSA and children with MRSA to those without carriage of *S. aureus*. The risk factors for MRSA colonization identified in the multivariate analysis are shown in Table 3. Overall, factors independently associated with an increased risk of MRSA colonization were different for male and female children and in various age groups. For male children, MRSA carriage was associated with antibiotic use in past 12 months (*p*<0.05, aged <1 year; *p*<0.01, aged 1–5 years; *p*<0.0001, aged >5 years), atopic dermatitis (*p*<0.001, aged <1 year; *p*<0.0001, aged 1–5 years and aged >5 years), and history of skin and soft tissue infection (*p*<0.01, aged <1 year and aged 1–5 years; *p*<0.05, aged >5 years). For male children aged <1 year only, chronic disease was associated with MRSA colonization (*p*<0.05). For male children aged 1–5 years, MRSA colonization was also associated with hospitalization in past 12 months (*p*<0.0001). For male children aged >5 years, household contact with hospital staff was associated with MRSA colonization (*p*<0.001).

**Table 3:** Risk factors associated with methicillin-resistant *Staphylococcus aureus* (MRSA) nasal colonization in children by multivariate analysis using polytomous logistic regression.

	MRSA Colonization, OR (95% CI)^a^					
	<1 year		1–5 years		>5 years	
	Male	Female	Male	Female	Male	Female
Risk factor	(n = 304)	(n = 273)	(n = 596)	(n = 627)	(n = 702)	(n = 698)
Personal risk factors						
Antibiotic use in						
past 12 months	4.1 (1.2–14.0)*		3.0 (1.5–6.1)**		12.3 (6.5–23.1)***	20.3 (10.1–40.8)***
Diagnosis of atopic						
dermatitis	7.6 (2.3–24.9)****		8.8 (4.0–19.5)***	9.7 (3.4–27.7)***	5.2 (2.4–11.3)***	5.0 (1.6–15.1)**
Chronic disease	23.0 (1.3–393.5)*					
Hospitalization in						
past 12 months			11.1 (5.5–22.3)***	5.9 (2.7–12.9)***		
History of skin/soft						
tissue infection	8.7 (1.7–44.4)**		3.1 (1.3–7.1)**	6.4 (2.6–15.5)***	1.9 (1.1–3.5)*	3.5 (1.8–6.8)****
Household risk factors						
Household contact						
with hospital staff		10.8 (2.0–58.3)**		4.4 (1.5–13.3)**	3.6 (1.7–7.7)****	

CI, confidence interval; OR, odds ratio.

a OR (95% CI) was presented as MRSA colonization vs. no. *S. aureus* colonization; *p* value of overall model was calculated by the Mantel-Haenszel test.

*p<0.05,

**p<0.01,

***p<0.0001,

****p<0.001.

The distribution of independent risk factors for MRSA carriage among female children in three different age groups was divergent. Among female children aged <1 year, household contact with hospital staff was associated with MRSA colonization (*p*<0.01). For female children aged 1–5 years, MRSA colonization was significantly associated with atopic dermatitis (*p*<0.0001), hospitalization in past 12 months (*p*<0.0001), history of skin and soft tissue infection (*p*<0.0001), and household contact with hospital staff (*p*<0.01). Among female children aged >5 years, antibiotic use in the past 12 months (*p*<0.0001), atopic dermatitis (*p*<0.01), and history of skin and soft tissue infection (*p*<0.001) were independently associated with MRSA colonization.

### Microbiologic characteristics of MRSA colonizing isolates

The prevalence and distribution of isolates associated with community transmission among MRSA colonized children are shown in Table 4. The prevalence of PVL and SCC*mec* V_T_ among MRSA colonizing isolates did not vary significantly with source, sex or age. However, comparison of the prevalence across surveys revealed that a significant increase occurred in 2007–2009 (*p*<0.0001). Among the 371 children colonized with MRSA, the largest proportion, 86.3% (95% CI, 82.8%–89.8%), carried ST59 clonal type. ST338 clonal type, a single-locus variant of ST59 (1 nucleotide difference in the *gmk* locus), was the next most prevalent MLST type and was recovered from 4.3% (95% CI, 2.3%–6.4%) of children with MRSA colonization. Children aged >5 years were more likely to be colonized with ST59 clonal type than those aged <1 year and 1–5 years (*p*<0.05). In addition, colonization with ST338 clonal type was significantly more prevalent in 2007–2009 compared with 2004–2006 (*p*<0.05), but this difference was not evident in ST59 clonal type.

**Table 4:** Prevalence of molecular traits among methicillin-resistant *Staphylococcus aureus* (MRSA) isolates recovered from MRSA-colonized children, by study variable, 2004-2009.

	Children colonized	No. (% [95% CI]) of isolates			
Variable	with MRSA, no.	PVL present	SCC*mec* V_T_ present	ST59	ST338
All participants	371	123 (33.2 [28.4–37.9])	101 (27.2 [22.7–31.8])	320 (86.3 [82.8–89.8])	16 (4.3 [2.3–6.4])
Source					
Health maintenance					
visits^a^	91	34 (37.4 [27.4–47.3])	29 (31.9 [22.3–41.4])	83 (91.2 [85.4–97.0])	5 (5.5 [0.8–10.2])
Kindergartens	280	89 (31.8 [26.3–37.2])	72 (25.7 [20.6–30.8])	237 (84.6 [80.4–88.9])	11 (3.9 [1.7–6.2])
Sex					
Male	198	65 (32.8 [26.3–39.4])	46 (23.2 [17.4–29.1])	169 (85.4 [80.4–90.3])	8 (4.0 [1.3–6.8])
Female^a^	173	58 (33.5 [24.5–40.6])	55 (31.8 [24.9–38.7])	151 (87.3 [82.3–92.3])	8 (4.6 [1.5–7.8])
Age, years					
<1^a^	45	18 (40.0 [25.7–54.3])	13 (28.9 [15.7–42.1])	34 (75.6 [63.0–88.1])	2 (4.4 [0.0–10.5])
1–5	123	42 (34.1 [25.8–42.5])	35 (28.5 [20.5–36.4])	102 (82.9 [76.3–89.6])	5 (4.1 [0.6–7.6])
>5	203	63 (31.0 [24.7–37.4])	53 (26.1 [20.1–32.2])	184 (90.6 [86.6–94.7]*)	9 (4.4 [1.6–7.3])
Survey					
2004–2006^a^	131	25 (19.1 [12.4–25.8])	14 (10.7 [5.4–16.0])	111 (84.7 [78.6–90.9])	1 (0.8 [0.0–2.3])
2007–2009	240	98 (40.8 [34.6–47.1]**)	87 (36.3 [30.2–42.3]**)	209 (87.1 [82.8–91.3])	15 (6.3 [3.2–9.3]*)

CI, confidence interval; PVL, Panton-Valentine leukocidin; SCC*mec*, staphylococcal cassette chromosome *mec*; ST, sequence type.

a Reference group.

*p<0.05,

**p<0.0001.

Except for an increase in the multidrug resistance rate in 2007–2009 (*p*<0.05), the resistance pattern, distribution of *erm* genotype and exotoxin profile of MRSA colonizing isolates did not vary significantly by source or survey period (Table 5 and Table 6). Although the resistance profile of ST59 clonal type did not change significantly during the study period (data not shown), isolates of ST338 clonal type expressed higher incidences of resistance to non-β-lactam antimicrobial agents (e.g., gentamicin and trimethoprim-sulfamethoxazole) and multiple drugs than isolates of ST59 clonal type (*p*<0.0001). Moreover, the combined results of molecular analyses showed that the majority (62.5%) of the 16 MRSA ST338 isolates expressed the genotypic profile ST338/SCC*mec* V_T_/PVL-positive/SEB-positive.

**Table 5:** Antimicrobial susceptibility for methicillin-resistant *Staphylococcus aureus* (MRSA) colonizing isolates in Taiwanese children, 2004-2009.

	Origin, no. (%)		Year, no. (%)		Clonal type, no. (%)			
Characteristic	H	K	2004–2006	2007–2009	ST59	ST338	*p* a	
Total isolates	91 (24.5)	280 (75.5)	131 (35.3)	240 (64.7)	320 (86.3)	16 (4.3)		
Resistance to								
Clindamycin	80 (87.9)	251 (89.6)	116 (88.5)	215 (89.6)	271 (84.7)	16 (100)	0.142	
Erythromycin	82 (90.1)	259 (92.5)	121 (92.4)	220 (91.7)	288 (90.0)	16 (100)	0.380	
Gentamicin	14 (15.4)	30 (10.7)	13 (9.9)	31 (12.9)	30 (9.4)	9 (56.3)	<0.0001	
TMP-SMX	2 (2.2)	5 (1.8)	0 (0)	7 (2.9)	2 (0.6)	5 (31.3)	<0.0001	
Ciprofloxacin	0 (0)	0 (0)	0 (0)	0 (0)	0 (0)	0 (0)		
Fusidic acid	0 (0)	0 (0)	0 (0)	0 (0)	0 (0)	0 (0)		
Mupirocin	1 (1.1)	0 (0)	0 (0)	1 (0.4)	1 (0.3)	0 (0)	0.999	
Rifampin	1 (1.1)	2 (0.7)	0 (0)	3 (1.3)	3 (0.9)	0 (0)	0.999	
Vancomycin	0 (0)	0 (0)	0 (0)	0 (0)	0 (0)	0 (0)		
Teicoplanin	0 (0)	0 (0)	0 (0)	0 (0)	0 (0)	0 (0)		
Multidrug^b^	14 (15.4)	36 (12.9)	11 (8.4)	39 (16.3)^c^	32 (10.0)	10 (62.5)	<0.0001	

H, Health maintenance visits; K, Kindergartens; ST, sequence type; TMP-SMX, trimethoprim-sulfamethoxazole.

a *p* value derived from comparison of isolates of ST59 and ST338 clonal type (chi-square test, unless otherwise indicated).

b Multiresistance to at least 3 of the 10 non-β-lactam antimicrobial agents tested.

c *p*<0.05 across surveys (2004–2006 vs. 2007–2009, Fisher's exact test).

**Table 6:** Origin, year and clonal type-specific characteristics of methicillin-resistant *Staphylococcus aureus* (MRSA) colonizing isolates in Taiwanese children.

	Origin, no. (%)		Year, no. (%)		Clonal type, no. (%)		
Characteristic	H	K	2004–2006	2007–2009	ST59	ST338	*p* a
Total isolates	91 (24.5)	280 (75.5)	131 (35.3)	240 (64.7)	320 (86.3)	16 (4.3)	
*erm* genotype, no. of							
strains/total no. of strains							
tested (% of isolates)							
*ermA*	3/82 (3.7)	13/259 (5.0)	6/121 (5.0)	10/220 (4.5)	13/288 (4.5)	0/16 (0)	0.999
*ermB*	79/82 (96.3)	246/259 (95.0)	115/121 (95.0)	210/220 (95.5)	275/288 (95.5)	16/16 (100)	0.999
Virulence factor							
ETA	2 (2.2)	11 (3.9)	3 (2.3)	10 (4.2)	11 (3.4)	0 (0)	0.999
ETB	4 (4.4)	8 (2.9)	5 (3.8)	7 (2.9)	9 (2.8)	1 (6.3)	0.390
TSST-1	11 (12.1)	29 (10.4)	13 (9.9)	27 (11.3)	33 (10.3)	0 (0)	0.383
SEA	6 (6.6)	18 (6.4)	10 (7.6)	14 (5.8)	19 (5.9)	1 (6.3)	0.999
SEB	76 (83.5)	235 (83.9)	104 (79.4)	207 (86.3)	269 (84.1)	14 (87.5)	0.999
SEC	3 (3.3)	10 (3.6)	4 (3.1)	9 (3.8)	7 (2.2)	0 (0)	0.999
SED	1 (1.1)	7 (2.5)	2 (1.5)	6 (2.5)	5 (1.6)	0 (0)	0.999
SEE	0 (0)	3 (1.1)	1 (0.8)	2 (0.8)	2 (0.6)	0 (0)	0.999
SEG/SEI	27 (29.7)	76 (27.1)	35 (26.7)	68 (28.3)	88 (27.5)	4 (25.0)	0.999
SEH	0 (0)	2 (0.7)	2 (1.5)	0 (0)	1 (0.3)	0 (0)	0.999

ETA, exfoliative toxin A; ETB, exfoliative toxin B; H, Health maintenance visits; K, Kindergartens; SEA, staphylococcal enterotoxin A; SEB, staphylococcal enterotoxin B; SEC, staphylococcal enterotoxin C; SED, staphylococcal enterotoxin D; SEE, staphylococcal enterotoxin E; SEG, staphylococcal enterotoxin G; SEH, staphylococcal enterotoxin H; SEI, staphylococcal enterotoxin I; ST, sequence type; TMP-SMX, trimethoprim-sulfamethoxazole; SST-1, toxic shock syndrome toxin-1.

a *p* value derived from comparison of isolates of ST59 and ST338 clonal type.

## Discussion

Several studies from the United States demonstrated that community-associated *S. aureus* infections among children and adults have increased rapidly in recent years and that MRSA is responsible for most of this increase [11], [41]. In Taiwan, CA-MRSA infections in pediatric patients have been noted since 2002 [13]–[16], [18], [42]. Subsequent to 2002, our pediatric infectious disease service has continued to see a dramatic increase in the number of pediatric consultations and evaluation for CA-MRSA infections in outpatient clinics, the pediatric emergency department and in pediatric wards. In addition, although the prevalence of MRSA colonization among children in the community has been extensively studied in Taiwan and the United States [14], [27], [43]–[48], few studies of MRSA colonization have examined the same population in a sequential manner [43]. In this study we attempted to characterize the MRSA colonization pool among children in the community served by our institute. Our results showed that the MRSA colonization rate among otherwise healthy children in community settings in Taiwan was 11.6% during the period from 2004 to 2009. The prevalence of *S. aureus* nasal colonization in this population was 25.8%, which is within the reported range and likely reflects that of the general population [43], [45], [48]–[52].

Between 2004–2006 and 2007–2009, the overall prevalence of *S. aureus* nasal colonization decreased, whereas the prevalence of MRSA colonization increased. These findings are consistent with a population-based study among people in the United States participating in the 2001 to 2004 National Health and Nutrition Examination Survey [49]. However, without additional surveillance it is impossible to determine whether these changes represent ongoing trends in the prevalence of colonization or are simply short-term modulations due to sampling variability or fluctuations in unmeasured variables including temperature and humidity [49]. Results from this study indicate that the prevalence of MRSA colonization in our community pediatric population in northern Taiwan was 8.1% during the period of 2004–2006, which is within the reported range (4.8%–9.5%) compared with a different population of healthy Taiwanese children in 2005 and 2006 [27]. However, the nasal MRSA colonization prevalence of healthy Taiwanese children from the present study increased significantly, from 7.3% during 2005–2006 to 15.1% (*p*<0.0001, chi-square test) during 2007–2009 [27]. There may be two reasons for this difference. First, the prevalence of MRSA colonization among children in communities may be higher in the northern region than in other geographic areas of Taiwan [27]. Second, the present study was conducted 3 years after the previous study, so the difference may be due to an overall increase of MRSA colonization prevalence during this time. Furthermore, the increasing trend in the prevalence of nasal MRSA colonization might account for the increasing incidence of CA-MRSA infection in Taiwanese children [13], [15], [18], [42], [53], [54].

Few studies have reported the determinants of MRSA colonization in community settings [55]. To the best of our knowledge, this is the first comprehensive survey of risk factors including personal and household risk factors for MRSA nasal carriage conducted in Taiwan, and it enrolled 3200 children including 2181 children attending 1 of 57 kindergartens in Taipei. Also, this is the first study in children to show that risk factors related to colonization with MRSA vary in terms of gender and age group. The multivariate analysis indicated that independent risk factors for MRSA colonization were use of antibiotics within the past year preceding sampling, diagnosis of atopic dermatitis, chronic disease, hospitalization in past 12 months, history of skin and soft tissue infection, and living with a family member who works in a hospital or clinic. These results have implications for pediatric infection control and clinical management strategies that are impacted by the different distribution of risk factors associated with an increase risk of MRSA colonization. For example, children with atopic dermatitis are more frequently colonized by MRSA in the anterior nares, which play a key role in the epidemiology and pathogenesis of concurrent skin and soft tissue infections [56].

It is well recognized that day care centers are efficient settings for the acquisition and transmission of pathogens and several determinants have been suggested, such as crowded conditions, frequent close physical contact, breakdown in appropriate hygiene and intensive exposure to antimicrobials [50]. Moreover, children with longer time periods of child care exposure are more prone to be colonized by MRSA than in children who are attending day care centers for the first time [24], [26]. An increase in MRSA colonization among children attending kindergartens in our study was anticipated, however, a decrease in nasal colonization with *S. aureus* was unexpected. These findings might be explained, in part, by the previously reported excessive use of antibiotics in Taiwan [57], [58], which highlights the importance of the problem of strong selective pressure from antimicrobial use in the community and may suppress MSSA and thus facilitate colonization by MRSA. In fact, it has been reported that Taiwanese physicians prescribe antibiotics to children under 16 years old for up to 45.5% of common colds [59]. We also found that the use of antibiotics was associated with MRSA colonization.

Previous studies throughout Taiwan found that ST59 MRSA isolates were the most common MLST type of MRSA causing CA-MRSA infections and nasal colonization [13]–[16], [27], [60]. Except in Western Australia and mainland China, however, ST59 MRSA isolates were rarely found in other Asian countries in recent large-scale studies [61]–[63]. In this study, molecular analysis indicated that the majority (90.6%) of MRSA colonizing isolates during the period 2004–2009 were clustered into minor variants of two clonal types, ST59 and ST338. Our results suggest that clonal spread of MRSA contributes to the high MRSA burden and that most colonized children acquired MRSA in the general community. The percentage of MRSA colonized children who carried ST59 with SCC*mec* IV/V_T_ was not significantly different between 2007–2009 (87.1%; 95% CI, 82.8%–91.3%) and 2004–2006 (84.7%; 95% CI, 78.6%–90.9%). Conversely, ST338 emerged in 2005 and accounted for 6.3% of MRSA colonizing isolates from 2007 to 2009 (*p*<0.05). More recently, PVL-positive CA-MRSA ST338 has been shown to be correlated with an increasing proportion of *S. aureus* infections among otherwise healthy children in the southern region of China and the majority of these clones belong to SCC*mec* V [63]. In the present study, most PVL-positive MRSA ST338 clones belonged to SCC*mec* V_T_, which contains a ccrC recombinase gene variant (ccrC2) and *mec* complex C2 [14]. We did not find any SCC*mec* V isolates, which may indicate that the background of ST338 clones in Taiwan is different from mainland China. In antibiotic susceptibility testing, MRSA colonizing isolates expressed high incidences of resistance to non-β-lactam antibiotics and multiple drugs. In a previous longitudinal study of 257 MRSA bloodstream infections, Chen et al. reported that multiresistance may have provided an advantage for the CA-MRSA strains to easily enter and rapidly proliferate in healthcare facilities, with resulting blurring of the distinction between CA-MRSA and healthcare-associated MRSA [12]. Therefore, the apparent increase in the multidrug resistance and transmissibility of MRSA colonizing isolates is cause for concern. Further investigation is needed to determine whether colonization with these strains is becoming more prevalent.

Several study limitations merit consideration. First, the survey design was cross-sectional and children who were only intermittently colonized may not have been detected. Thus, the frequency of colonization with organisms might have been underestimated. Second, recall bias may have occurred when parents or guardians attempted to remember past events and exposures assessed by the questionnaire. In addition, the relatively small number of children less than 1 year of age with MRSA colonization might have limited the ability to detect all risk factors. Third, the persistence of MRSA colonization in the study subjects could not be determined and the incidence of subsequent MRSA infection could not be measured. Finally, this study was conducted at a single site and may not reflect colonization rates throughout the country.

In conclusion, this sequential, prospective observational study found the prevalence of MRSA nasal colonization increased in Taiwanese children from 2004–2006 to 2007–2009, despite a decrease in the overall prevalence of *S. aureus* colonization during the same period. Our investigation provides evidence of the constantly changing epidemiology of staphylococcal colonization in our community. Moreover, the detection of 16 isolates belonging to ST338, a multiresistant clone frequently associated with PVL genes, is of special concern in a young population as it has the potential to sharply limit therapeutic options should it become widespread.
